# Absence of *Wolbachia *endobacteria in the non-filariid nematodes *Angiostrongylus cantonensis *and *A. costaricensis*

**DOI:** 10.1186/1756-3305-1-31

**Published:** 2008-09-18

**Authors:** Jeremy M Foster, Sanjay Kumar, Louise Ford, Kelly L Johnston, Renata Ben, Carlos Graeff-Teixeira, Mark J Taylor

**Affiliations:** 1 Parasitology Division, New England Biolabs, 240 County Road, Ipswich, MA 01938, USA; 2 Molecular and Biochemical Parasitology, Liverpool School of Tropical Medicine, Pembroke Place, Liverpool L3 5QA, UK; 3 Laboratório de Parasitologia Molecular, Instituto de Pesquisas Biomédicas da PUCRS, Porto Alegre, Brazil

## Abstract

The majority of filarial nematodes harbour *Wolbachia *endobacteria, including the major pathogenic species in humans, *Onchocerca volvulus*, *Brugia malayi *and *Wuchereria bancrofti*. These obligate endosymbionts have never been demonstrated unequivocally in any non-filariid nematode. However, a recent report described the detection by PCR of *Wolbachia *in the metastrongylid nematode, *Angiostrongylus cantonensis *(rat lungworm), a leading cause of eosinophilic meningitis in humans. To address the intriguing possibility of *Wolbachia *infection in nematode species distinct from the Family Onchocercidae, we used both PCR and immunohistochemistry to screen samples of *A. cantonensis *and *A. costaricensis *for the presence of this endosymbiont. We were unable to detect *Wolbachia *in either species using these methodologies. In addition, bioinformatic and phylogenetic analyses of the *Wolbachia *gene sequences reported previously from *A. cantonensis *indicate that they most likely result from contamination with DNA from arthropods and filarial nematodes. This study demonstrates the need for caution in relying solely on PCR for identification of new endosymbiont strains from invertebrate DNA samples.

## Findings

*Wolbachia *endobacteria infect most species of insect and are present in other arthropod groups as well as in most filarial nematode species. Phylogenetic analyses currently indicate as many as eight distinct *Wolbachia *lineages, designated supergroups A to H, along with some other lineages whose taxonomic position remains unresolved [1,2]. Arthropod *Wolbachia *are found in all supergroups except C and D, with the majority of insect *Wolbachia *strains in supergroups A and B. *Wolbachia *from filarial nematodes are exclusively in supergroups C and D with the exception of endosymbionts from *Mansonella spp*., which are in supergroup F along with the *Wolbachia *from certain termites.

The association between *Wolbachia *and filarial nematodes appears to be one of mutualism, probably of an obligatory nature [3]. Elimination of the endosymbionts by antibiotic treatment disrupts embryogenesis and hence microfilarial production, disrupts growth and development, and leads to macrofilaricidal effects. *Wolbachia *are also implicated in the immunopathology of infected persons and may contribute to the inflammatory adverse events seen after standard anti-filarial chemotherapy [3]. Thus, targeting the *Wolbachia *endosymbiont has emerged as an attractive new strategy for filarial disease control [3].

Until recently, despite extensive investigation of diverse nematode groups, there had been no identification of *Wolbachia *in any non-filariid nematode [4,5]. However, *Wolbachia ftsZ*, *wsp *(*Wolbachia *surface protein) and 16S rDNA sequences were recently amplified by PCR from DNA preparations of the metastrongylid nematode, *Angiostrongylus cantonensis *[6]. Based on phylogenetic analysis of the *wsp *sequence, the apparent endosymbiont from *A. cantonensis *appeared to have a lineage distinct from the filarial *Wolbachia *(supergroups C, D or F) and was tentatively positioned in supergroup G, containing the *Wolbachia *from certain spiders such as *Diaea circumlita*. Both *A. cantonensis *and *A. costaricensis *are occasional pathogens of humans, the former a leading cause of eosinophilic meningitis in Asia and Pacific Islands while the latter produces abdominal disease in the Americas [7,8]. The unexpected detection of *Wolbachia *in *A. cantonensis *and the medical implications of targeting this endosymbiont for novel antibiotic therapies for control of eosinophilic meningitis prompted us to investigate the status of *Wolbachia *in the genus *Angiostrongylus *in more detail.

*A. cantonensis *was obtained from Prof. Kentaro Yoshimura and maintained as a laboratory life-cycle in the Department of Parasitology of the Akita University Medical School. *A. costaricensis *strain "Santa Rosa" has been maintained in the Laboratório de Parasitologia Molecular, Instituto de Pesquisas Biomédicas da PUCRS since 1992 (in mice and the wild rodent *Oligoryzomis nigripes*; veronicelid slugs and *Biomphalaria glabrata *snails). Adult *Brugia malayi *(TRS labs) were used as a positive control for PCR and immunohistochemistry

PCR analysis of *Wolbachia *genes from both *A. cantonensis *and *A. costaricensis *adult worms showed no evidence that either species harbours *Wolbachia *endosymbionts. Genomic DNA from individual adult female *A. cantonensis *and *A. costaricensis*, which had been stored in 80% ethanol, was isolated (QiaAmp^® ^DNA mini kit, Qiagen) and analysed for *Wolbachia *by PCR as previously described [6] with modifications. PCR was carried out on a iCycler thermocycler (Bio-Rad) using the following conditions: 95°C for 4 min, followed by 40 cycles of 94°C for 15 s, 48°C for 30 s, 72°C for 2 min, then 72°C for 10 min, using primer pairs widely used for detection of *Wolbachia *from diverse hosts, and previously used on DNA from *A. cantonensis *[6]. The following primers were used for amplification of *Wolbachia *gene sequences: *wsp *(wsp81F; 5'-TGG TCC AAT AAG TGA TGA AGA AAC-3' and wsp691R; 5'-AAA AAT TAA ACG CTA CTC CA-3'); *ftsZ *(ftsZ357F; 5'-CAA AAA TAT GTG GAT ACG CTC ATT GT-3' and ftsZ788R; 5'-GTA GCA CCA AAT ATT ATA TTT GCA TTT TC-3'); and 16S rRNA (16SwolbF; 5'-GAA GAT AAT GAC GGT ACT CAC-3' and 16SwolbR3; 5'-GTC ACT GAT CCC ACT TTA AAT AAC-3'). PCR reactions were performed in 25 μl containing 1 μl gDNA, 0.3 μM of each primer, 0.2 mM dNTPs, 1.5–3.0 mM MgCl_2 _and 0.625 U of Taq polymerase (New England Biolabs [NEB]) in 1× reaction buffer (NEB). Positive PCR amplification was shown using DNA isolated from individual adult female *B. malayi*, which are known to contain *Wolbachia*, thus demonstrating that the primers and conditions were optimal for *Wolbachia *detection. All DNA samples produced nematode specific gene products. *Angiostrongylus *18S rRNA, based on GenBank sequences from *A. cantonensis *and *A. costaricensis *([GenBank:AY295804] and [GenBank:EF514913], respectively) was amplified with primers Ac18S 30F; 5'-AAG TGA AAC TGC GAA CGG CT-3' and Ac18S 830R; 5'-TCA CCT CTC GCG CAG GGA TA-3', while *B. malayi gst *was amplified as previously described [9] using GST 1377; 5'-TGC TCG CAA ACA TAG TAA TAG T-3' and GST 1632; 5'-ATC ACG GAC GCC TTC ACA G-3', indicating that there was DNA at sufficient concentrations for detection by standard one-round PCR.

In order to detect *Wolbachia *by immunohistochemistry, *A. cantonensis *and *A. costaricensis *worms were fixed in 80% ethanol and embedded in paraffin blocks. Sections were stained using affinity purified anti-*Wolbachia *peptidoglycan-associated lipoprotein (WoLP) antibodies and rabbit polyclonal anti-sera raised to *Wolbachia *surface protein (WSP) and visualized using the UltraVision ONE detection system (Lab Vision, ThermoFisher Scientific) using haematoxylin as a counterstain. In contrast to the positive staining observed in sections of *B. malayi*, both *Angiostrongylus *species were found to be negative with each of the *Wolbachia*-specific antibodies or antisera. It should be noted that both of these reagents are able to detect *Wolbachia *in species as diverse as filarial nematodes and the mosquito *Aedes albopictus *[[9-11], M. Taylor, unpublished data], indicating that a lack of cross-reactivity to *Wolbachia *found in *Angiostrongylus *is unlikely.

Because we were unable to reproduce the detection of *Wolbachia *in *Angiostrongylus spp *by PCR or immunohistochemistry, we analyzed in detail the sequences deposited in the GenBank database as part of the earlier report [6]. Comparison of the *wsp *nucleotide sequence [GenBank:AY508980] to the non-redundant nucleotide database (nr) using BLASTN revealed good identity (97%) to the *wsp *sequence [GenBank:AY486092] from the putative supergroup G *Wolbachia *from the spider, *D. circumlita *c2, as described previously [6]. However, the best identity (99%) was to *wsp *from the *Wolbachia *of the mosquito, *Malaya genurostris *[GenBank:AY462865]. *Wolbachia *multilocus sequence typing (MLST) has shown that phylogenetic inference based upon *wsp *sequences yields spurious lineages due to the high levels of intragenic recombination in this gene [1]. MLST of the *Wolbachia *of the spider, *D. circumlita *c2, indicates that this endosymbiont is more correctly a member of supergroup A [1], and so too presumably is the *Wolbachia *from the mosquito, *M. genurostris*.

**Figure 1:**
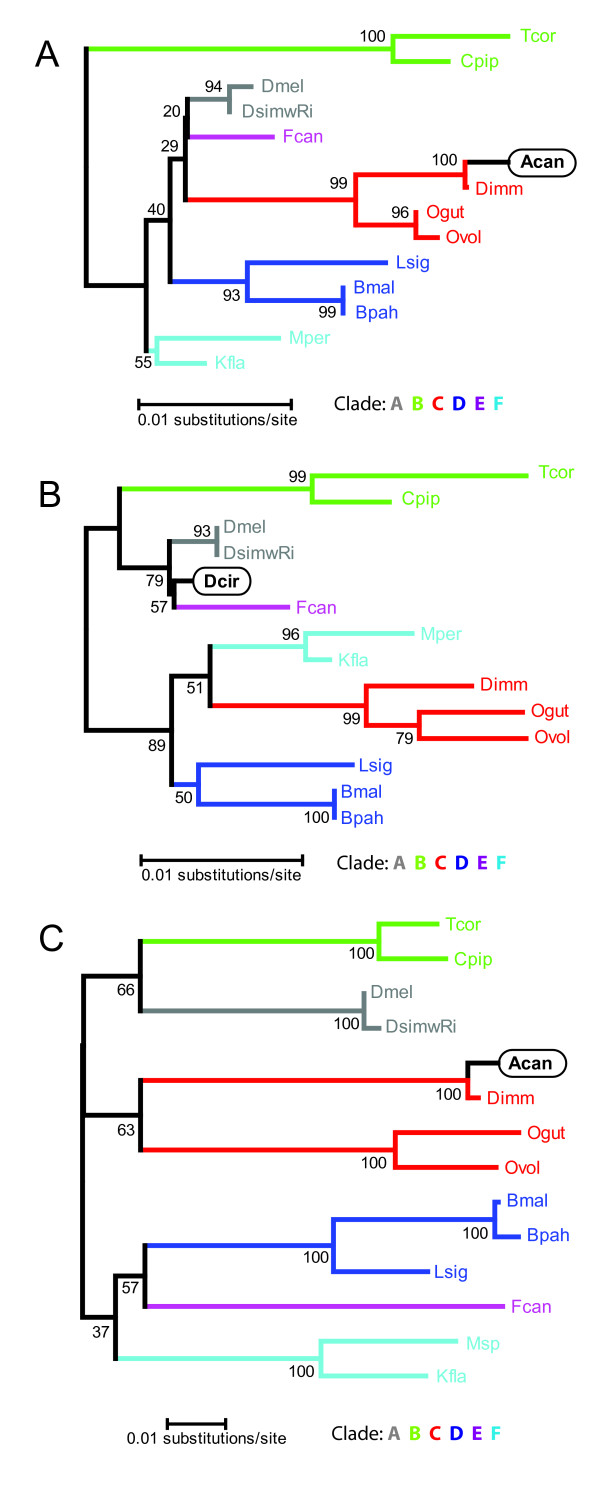
**Minimum evolution trees based on alignments of A) the *****Wolbachia*****16S (770 nucleotides) reported from***** A**. **can****tonensis*****[GenBank:**AY652762], **B) the *Wolbachia *16S (639 nucleotides) of *D. circumlita *c2 [GenBank:**AY486072]**, and C) the *Wolbachia ftsZ *(431 nucleotides) reported from *A. cantonensis *[GenBank:**DQ159068** ]**. Sequences were aligned using ClustalX version 2.0.7 [14] using default parameters for slow/accurate alignment [Gap Opening:10, Gap Extend: 0.1, IUB DNA weight matrix]. After alignment, sequences were manually trimmed to the endpoints of the 16S sequences of the *Wolbachia *from *A. cantonensis *(see additional file 1: *Wolbachia *16S multiple sequence alignment – *A. cantonensis*) and *D. circumlita *(see additional file 2: *Wolbachia *16S multiple sequence alignment – *D. circumlita*), and to the endpoints of the *ftsZ *sequence of the *Wolbachia *from *A. cantonensis *(see additional file 3: *Wolbachia *ftsZ multiple sequence alignment). Phylogenetic trees were calculated using the Minimum Evolution method in MEGA4 [15]. The percentage of replicate trees in which the associated taxa clustered together in the bootstrap test (1000 replicates) is shown next to the branches. Evolutionary distances were computed using the Maximum Composite Likelihood. The Minimum Evolution tree was searched using the Close-Neighbor-Interchange (CNI) algorithm at a search level of 1. The Neighbor-joining algorithm was used to generate the initial tree. All positions containing gaps and missing data were eliminated from the dataset (Complete deletion option).

We performed similar nucleotide comparisons of the 16S [GenBank:AY652762] and *ftsZ *[GenBank:DQ159068] sequences attributed to *Wolbachia *from *A. cantonensis *and constructed phylogenetic trees for each. *Wolbachia *sequences used for multiple alignments and phylogenetic tree construction are as follows, with the first GenBank sequence for each invertebrate host organism corresponding to 16S and the second to *ftsZ*: *Brugia malayi *[GenBank:AJ010275], [GenBank:AJ010269]; *B. pahangi *[GenBank:AJ012646], [GenBank:AJ010270]; *Litomosoides sigmodontis *[GenBank:AF069068], [GenBank:AJ010271]; *Onchocerca volvulus *[GenBank:CU062464], GenBank:AJ276501]; *O. gutturosa *[GenBank:AJ276498], [GenBank:AJ010266]; *Dirofilaria immitis *[Genbank:Z49261], [GenBank:AJ010272]; *Drosophila melanogaster *[GenBank: NC_002978.6; genome coordinates: 1167943–1169389], [GenBank:U28189]; *D. simulans w*Riverside [GenBank:DQ412085], [GenBank:U28178]; *Trichogramma cordubensis *[GenBank:L02883], [GenBank:U28200]; *Culex pipiens *[Genbank:U23709], [GenBank:U28209]; *Folsomia candida *[GenBank:AF179630], [GenBank:AJ344216]; *Kalotermes flavicollis *[GenBank:Y11377], [GenBank:AJ292345], *Mansonella perstans *16S [GenBank:AY278355]; *Mansonella sp*. *ftsZ *[GenBank:AJ628414]. The underlined characters for each host species represent the abbreviations shown in the trees. The 16S sequence attributed to *Wolbachia *from *A. cantonensis *had highest nucleotide identity (99%) to *Wolbachia *16S from *D. immitis *[GenBank:Z49261]. This identity was better than that between *Wolbachia *16S sequences from sister species within the genus *Dirofilaria*, namely *D. immitis *and *D. repens *(98%). An equal match was detected for *Wolbachia *apparently from an engorged dog tick, *Rhipicephalus sanguineus *[GenBank:AF304445], but since this tick lacks *Wolbachia *[12], we propose that this *Wolbachia *sequence derived from *D. immitis *acquired during a blood feed on a heartworm-infected dog. The 16S phylogenetic tree resolved supergroups A to F and confirmed the near identity of the sequence amplified from *A. cantonensis *to the *Wolbachia *16S from *D. immitis *(Figure 1a; see additional file 1: *Wolbachia *16S multiple sequence alignment – *A. cantonensis*). The available 16S sequences for *Wolbachia *from *A. cantonensis *and from *D. circumlita *are partial gene sequences and have very little overlap with each other. Therefore, we were unable to include *D. circumlita Wolbachia *16S in the same alignment and phylogenetic tree as the 16S reported from *Wolbachia *from *A. cantonensis*. However, a separate alignment and tree using 16S fragments corresponding to that from *Wolbachia *of *D. circumlita *showed that this sequence is quite distinct from the *D. immitis Wolbachia *16S and clusters with sequences from *Wolbachia *of arthropods (Figure 1b; see additional file 2: *Wolbachia *16S multiple sequence alignment – *D. circumlita*), as expected based on recent *Wolbachia *MLST [1]. Therefore, while the *wsp *gene reported for *Wolbachia *from *A. cantonensis *has high identity to sequences from the endosymbionts of the arthropods, *M. genurostris *and *D. circumlita*, the 16S sequence is nearly identical to *Wolbachia *16S from filarial nematodes, notably *D. immitis *(supergroup C). The results of our analyses of *ftsZ *were very similar to those obtained for 16S. We observed 99% identity to the *ftsZ* reported from the *Wolbachia *from *D. immitis *[GenBank:AJ010272]. The level of identity between these two sequences also exceeded that between the *Wolbachia ftsZ *sequences from the sister species *D. immitis *and *D. repens *(92%). A phylogenetic analysis of *ftsZ *sequences representing *Wolbachia *supergroups A to F (Figure 1c; see additional file 3: *Wolbachia ftsZ* multiple sequence alignment) also resolved these six groups and confirmed the high similarity of the sequence reported for *Wolbachia *from *A. cantonensis *and that from *D. immitis*.

In conclusion, our inability to detect *Wolbachia *in two different species of *Angiostrongylus *by PCR or immunohistochemistry argues against the presence of this endosymbiont in these metastrongylid nematodes. Lateral gene transfers from *Wolbachia *to invertebrates are common [13]. Such a phenomenon could conceivably have resulted in the presence of *Wolbachia *fragments in the *A. cantonensis *genome, but since we were unable to detect *Wolbachia *sequences using the same PCR primers as were used in the earlier report [6], this possibility seems most unlikely. Instead, we suspect contamination of the DNA samples or PCR reactions with *Wolbachia *DNA from the mosquito, *M. genurostris *in the case of *wsp *and the filarial nematode, *D. immitis*, in the case of both *ftsZ *and 16S. This seems very plausible since the nucleotide identity is 99% in all cases. In support of this conclusion, we note that the *wsp *sequence of the *M. genurostris *endosymbiont was deposited in the GenBank database by the same authors of the recent report on *Wolbachia *in *A. cantonensis*, and that they carry out research on *D. immitis*. We cannot rule out the possibility that *A. cantonensis *from Taiwan contain *Wolbachia *while the worms we analyzed from Japan do not. However, the high identities of the reported sequences to those from arthropod *Wolbachia *(supergroup A) on the one hand, but to the *Wolbachia *from the nematode, *D. immitis *(supergroup C), on the other, evoke either a double *Wolbachia *infection, a phenomenon never observed in nematode-*Wolbachia *symbioses, or a highly divergent *Wolbachia *lineage unlike any reported thus far. The most straightforward conclusion from our analysis is that *Angiostrongylus sp*. do not contain *Wolbachia*.

## Competing interests

The authors declare that they have no competing interests.

## Authors' contributions

MT and JF wrote the paper. JF and SK performed bioinformatic analyses and constructed the multiple sequence alignments and phylogenetic trees. LF carried out PCR analyses and KJ immunohistochemistry and both contributed to writing the paper. RB and C G-T performed the initial PCR analysis and provided parasite material. MT conceived and directed the study. All authors read and approved the final manuscript.

## Supplementary Material

Additional file 1 *Wolbachia *16S multiple sequence alignment – *A. cantonensis*. A multiple sequence alignment of the 16S sequence attributed to *Wolbachia *from *A. cantonensis *and corresponding 16S fragments from *Wolbachia *from diverse arthropod and nematode hosts. Click here for file

Additional file 2 *Wolbachia *16S multiple sequence alignment – *D. circumlita*. A multiple sequence alignment of the 16S sequence of *Wolbachia *from *D. circumlita *and corresponding 16S fragments from *Wolbachia *from diverse arthropod and nematode hosts. Click here for file

Additional file 3 *Wolbachia ftsZ *multiple sequence alignment. A multiple sequence alignment of the *ftsZ *sequence attributed to *Wolbachia *from *A. cantonensis *and corresponding *ftsZ *fragments from *Wolbachia *from diverse arthropod and nematode hosts. Click here for file
