# Supplementary material for: Absence of Wolbachia endobacteria in the non-filariid nematodes Angiostrongylus cantonensis and A. costaricensis
Source: Parasit Vectors. 2008 Sep 18;1:31. doi: 10.1186/1756-3305-1-31 (PMC2565651; doi:10.1186/1756-3305-1-31)
Supplement: Additional file 2 — Wolbachia 16S multiple sequence alignment – D. circumlita. A multiple sequence alignment of the 16S sequence of Wolbachia from D. circumlita and corresponding 16S fragments from Wolbachia from diverse arthropod and nematode hosts. [file 1756-3305-1-31-S2.pdf]

```
          10        20        30        40        50        60        70        80        90
Bmal      GGAAACGGCAACTAATACCGTATACGCCCTACGGGGGAAAGATTTATTGCTATTAGATGAGCCTATATTAGATTAGC-TAGTTGGTAGGGTAATGGCCTA
Ovol      GGAAACGACAACTAATACCGTATACGCCCTACGGGGGAAAGATTTATTGCTATTGGATGAGCCTATATTAGATTAGC-TAGTTGGTAGGGTAATGGCCTG
Ogut      GGAAACGACAACTAATACCGTATACGCCCTACGGGGGAAAGATTTATTGCTATTAGATGAGCCTATATTAGATTAGC-TAGTTGGTAGGGTAATGGCCTA
Dimm      GGAAACGGCAACTAATACCGTATACGCCCTATGGGGGAAAGATTTATTGCTATTAGATGAGCCTATATTAGATTAGC-TAGTTGGTAGGGTAATGGCCTA
Lsig      GGAAACGGCAACTAATACCGTATACGCCCTACGGGGGAAAGATTTATTGCTATTAGATGAGCCTATATTAGATTAGC-TAGTTGGTGGGGTAATAGCTTA
Mper      GGAAACGGCAACTAATACCGTATACGCCCTACGGGGGAAAGATTTATTGCTATTAGATGAGCCTATATTAGATTAGC-TAGTTGGTAAGGTAATGGCTTA
Bpah      GGAAACGGCAACTAATACCGTATACGCCCTACGGGGGAAAGATTTATTGCTATTAGATGAGCCTATATTAGATTAGC-TAGTTGGTAGGGTAATGGCCTA
Kfla      GGAAACGGCAACTAATACCGTATACGCCCTACGGGGGAAAGATTTATTGCTATTAGATGAGCCTATATTAGATTAGC-TAGTTGGTAAGGTAATGGCTTA
Fcan      GGAAACGGCAACTAATACCGTATACGCCCTACGGGGGAAAAATTTATTGCTATTAGATGAGCCTATATTAGATTAGC-TTGTTGGTGGGGTAATGGCCTA
Dmel      GGAAACGGCAACTAATACCGTATACGCCCTACGGGGGAAAAATTTATTGCTATTAGATGAGCCTATATTAGATTAGC-TAGTTGGTGGAGTAATAGCCTA
DsimwRi   GGAAACGGCAACTAATACCGTATACGCCCTACGGGGGAAAAATTTATTGCTATTAGATGAGCCTATATTAGATTAGC-TAGTTGGTGGAGTAATAGCCTA
Tcor      GGAAACGGCAACTAATACCGTATACGCCCTATGGGGGAAAAATTTATTGCTATTAGATGAGCCTATATTAGATTAGC-TAGTTGGTGGAGTAATAGCCTA
Cpip      GGAAACGACAACTAATACCGTATACGCCCTAC-GGGGAAAAATTTATTGCTATTAGATGAGCCTATATTAGATTAGC-TAGTTGGTGGGGTAATAGCCTA
Dcir      GGAAACGGCAACTAATACCGTATACGCCCTACGGGGGAAAAA-TTATTGCTATTAGATGAGCCTATATTAGATTAGCTTAGTTGGTGGGGTAATGGCCTA

          110       120       130       140       150       160       170       180       190
Bmal      CCAAGGCAGTAATCTATAGCTGATCTGAGAGGATGATCAGCCACACTGGAACTGAGATACGGTCCAGACTCCTACGGGAGGCAGCAGTGGGGAATATTGG
Ovol      CCAAGGCTATGATCTATAGCTGATCTGAGAGGATGGTCAGCCACACTGGAACTGAGATACGGTCCAGACTCCTACGGGAGGCAGCAGTGGGGAATATTGG
Ogut      CCAAGGCTATGATCTATAGCTGATCTGAGAGGATGATCAGCCACACTGGAACTGAGATACGGTCCAGACTCCTACGGGAGGCAGCAGTGGGGAATATTGG
Dimm      CCAAGGCTATGATCTATAGCTGATCTGAGAGGATGATCAGCCACACTGGAACTGAGATACGGTCCAGACTCCTACGGGAGGCAGCAGTGGGGAATATTGG
Lsig      CCAAGGCAATGATCTATAGCTGATCTGAGAGGATGATCAGCCACACTGGAACTGAGATACAGTCCAGACTCCTACGGGAGGCAGCAGTGGGGAATATTGG
Mper      CCAAGGCAATGATCTATAGCTGATCTGAGAGGATGATCAGCCACACTGGAACTGAGACACGGTCCAGACTCCTACGGGAGGCAGCAGTGGGGAATATTGG
Bpah      CCAAGGCAGTAATCTATAGCTGATCTGAGAGGATGATCAGCCACACTGGAACTGAGATACGGTCCAGACTCCTACGGGAGGCAGCAGTGGGGAATATTGG
Kfla      CCAAGGCAATGATCTATAGCTGATCTGAGAGGATGATCAGCCACACTGGAACTGAGATACGGTCCAGACTCCTACGGGAGGCAGCAGTGGGGAATATTGG
Fcan      CCAAGGCAATGATCTATAGCTGATCTGAGAGGATGATCAGCCACACTGGAACTGAGATACGGTCCAGACTCCTACGGGAGGCAGCAGTGGGGAATATTGG
Dmel      CCAAGGCAATGATCTATAGCTGATCTGAGAGGATGATCAGCCACACTGGAACTGAGATACGGTCCAGACTCCTACGGGAGGCAGCAGTGGGGAATATTGG
DsimwRi   CCAAGGCAATGATCTATAGCTGATCTGAGAGGATGATCAGCCACACTGGAACTGAGATACGGTCCAGACTCCTACGGGAGGCAGCAGTGGGGAATATTGG
Tcor      CCAAGGCAATGATCTATAGCTGATCTGAGAGGATGATCAGCCACACTGGAACTGAGATACGG-CCAGACTCCTACGGGAGGCAGCAGTGGGGAATATTGG
Cpip      CCAAGGTAATGATCTATAGCTGATCTGAGAGGATGATCAGCCACACTGGAACTGAGATACGGTCCAGACTCCTACGGGAGGCAGCAGTGGGGAATATTGG
Dcir      CCAAGGCAATGATCTATAGCTGATCTGAGAGGATGATCAGCCACACTGGAACTGAGATACGGTCCAGACTCCTACGGGAGGCAGCAGTGGGGAATATTGG

          210       220       230       240       250       260       270       280       290
Bmal      ACAATGGGCGAAAGCCTGATCCAGCCATGCCGCATGAGTGAAGAAGGCCTTTGGGTTGTAAAGCTCTTTCAGTGAGGAAGATAATGACGGTACTCACAGA
Ovol      ACAATGGGCGAAAGCTTGATCCAGCCATGCCGCGTGAGTGAAGAAGGCCTTTGGGTTGTAAAGCTCTTTCAGTGAGGAAGATAATGACGGTACTCACAGA
Ogut      ACAATGGGCGGAAGCTTGATCCAGCCATGCCGCGTGAGTGAAGAAGGCCTTTGGGTTGTAAAGCTCTTTCAGTGAGGAAGATAATGACGGTACTCACAGA
Dimm      ACAATGAGCGGAAGCTTGATCCAGCTATGCCGCATGAGTGAAGAAGGCCTTTGGGTTGTAAAGCTCTTTCAGTGAGGAAGATAATGACGGTACTCACAGA
Lsig      ACAATGGGCGAAAGCCTGATCCAGCCATGCCGCATGAGTGAAGAAGGCCTTTGGGTTGTAAAGCTCTTTCAGTGAGGAAGATAATGACGGTACTCACAGA
Mper      ACAATGGGCGAAGGCCTGATCCAGCCATGCCGCATGAGTGAAGAAGGCCTTTGGGTTGTAAAGCTCTTTCGGTGAGGAAGATAATGACGGTACTCACAGA
Bpah      ACAATGGGCGAAAGCCTGATCCAGCCATGCCGCATGAGTGAAGAAGGCCTTTGGGTTGTAAAGCTCTTTCAGTGAGGAAGATAATGACGGTACTCACAGA
Kfla      ACAATGGGCGAAAGCCTGATCCAGCCATGCCGCATGAGTGAAGAAGGCCTTTGGGTTGTAAAGCTCTTTCGGTGAGGAAGATAATGACGGTACTCACAGA
Fcan      ACAATGGGCGAAAGCCTGATCCAGCCATGCCGCATGAGTGAAGAAGGCCTTCGGGTTGTAAAGCTCTTTTAGTGAGGAAGATAATGACGGTACTCACAGA
Dmel      ACAATGGGCGAAAGCCTGATCCAGCCATGCCGCATGAGTGAAGAAGGCCTTTGGGTTGTAAAGCTCTTTTAGTGAGGAAGATAATGACGGTACTCACAGA
DsimwRi   ACAATGGGCGAAAGCCTGATCCAGCCATGCCGCATGAGTGAAGAAGGCCTTTGGGTTGTAAAGCTCTTTTAGTGAGGAAGATAATGACGGTACTCACAGA
Tcor      ACAATGGGCGAAANNNTGATCCAGCCATGCCGCATGAGTGAAGAAGGCCTTTGGGTTGTAAAGCTCTTTTAGCGAGGAAGATGATGACGGTACTCACTGA
Cpip      ACAATGGGCGAAAGCCTGATCCAGCCATGCCGCATGAGTGAAGAAGGCCTTTGGGTTGTAAAGCTCTTTTAGTGAGGAAGATAATGACGGTACTCACAGA
Dcir      ACAATGGGCGAAAGCTTGATCCAGCCATGCCGCATGAGTGAAGAAGGCCTTTGGGTTGTAAAGCTCTTTTAGTGAGGAAGATAATGACGGTACTCACAGA

          310       320       330       340       350       360       370       380       390
Bmal      AGAAGTCCTGGCTAACTCCGTGCCAGCAGCCGCGGTAATACGGAGAGGGCTAGCGTTATTCGGAATTATTGGGCGTAAAGGGCGCGTAGGCTGATTAGTA
Ovol      AGAAGTCCTGGCTAACTCCGTGCCAGCAGCCGCGGTAATACGGAGAGGGCTAGCGTTATTCGGAATTATTGGGCGTAAAGAGCACGTAGGCTGGTTAGTA
Ogut      AGAAGTCCTGGCTAACTCCGTGCCAGCAGCCGCGGTAATACGGAGAGGGCTGCCGTTATTCGGAATTATTGGGCGTAAAGAGCACGTAGGCTGGTTAGTA
Dimm      AGAAGTCCTGGCTAACTCCGTGCCAGCAGCCGCGGTAATACGGAGAGGGCTAGCGTTATTCGGAATTATTGGGCGTAAAGAGCACGTAGGCTGGTTAGTA
Lsig      AGAAGTCCTGGCTAACTCCGTGCCAGCAGCCGCGGTAATACGGAGAGGGCTAGCGTTATTCGGAATTATTGGGCGTAAAGGGCGCGTAGGCTGATTAGTA
Mper      AGAAGTCCTGGCTAACTCCGTGCCAGCAGCCGCGGTAATACGGAGAGGGCTAGCGTTATTCGGAATTATTGGGCGTAAAGAGCGCGTAGGCCGGTTAGTA
Bpah      AGAAGTCCTGGCTAACTCCGTGCCAGCAGCCGCGGTAATACGGAGAGGGCTAGCGTTATTCGGAATTATTGGGCGTAAAGGGCGCGTAGGCTGATTAGTA
Kfla      AGAAGTCCTGGCTAACTCCGTGCCAGCAGCCGCGGTAATACGGAGAGGGCTAGCGTTATTCGGAATTATTGGGCGTAAAGAGCGCGTAGGCTGGTTAGTA
Fcan      AGAAGTCCTGGCTAACTCCGTGCCAGCAGCCGCGGTAATACGGAGAGGGCTAGCGTTATTCGGAATCATTGGGCGTAAAGGGCGCGTAGGCGGATTAGTA
Dmel      AGAAGTCCTGGCTAACTCCGTGCCAGCAGCCGCGGTAATACGGAGAGGGCTAGCGTTATTCGGAATTATTGGGCGTAAAGGGCGCGTAGGCGGATTAGTA
DsimwRi   AGAAGT-CTGGCTAACTCCGTGCCAGCAGCCGC-GTAATACGGAGAGGGCTAGCGTTATTCGGAATTATTGGGCGTAAAGGGCGCGTAGGCTGGTTAATA
Cpip      AGAAGTCCTGGCTAACTCCGTGCCAGCAGCCGCGGTAATACGGAGAGGGCTAGCGTTATTCGGAATTATTGGGCGTAAAGGGCGCGTAGGCTGGTTAATA
Dcir      AGAAGTCCTGGCTAACTCCGTGCCAGCAGCCGCGGTAATACGGAGAGGGCTAGCGTTATTCGGAATTATTGGGCGTAAAGGGCGCGTAGGCGGATTAGTA

          410       420       430       440       450       460       470       480       490
Bmal      AGTTAAAAGTGAAATCCCAAAGCTTAACTTTGGAATTGCTTTTAAAACTGTTAATCTAGAGGTTGAAAGAGGATAGAGGAATTCCTAGTGTAGAGGTGAT
Ovol      AGTTAAAAGTGAAATTCCAAAGCTTAACTTTGGAATTGCTTTTAAAACTGCTGATCTAGAGGTTGAAAGAGGATAGAGGAATTCCTAGTGTAGAGGTGAA
Ogut      AGTTAAAAGTGAAATTCCAAAGCTTAACTTTGGAATTGCTTTTAAAACTGCTGACCTAGAGATTGAAAGAGGATAGAGGAATTCCTAGTGTAGAGGTGAA
Dimm      AGTTAAAAGTGAAATTCCAAAGCTTAACTTTGGAATTGCTTTTAAAACTGCTGATCTAGAGATTGAAAGAGGATAGAGGAATTCCTAGTGTAGAGGTGAA
Lsig      AGTTAAGAGTGAAATCCCAAAGCTCAACTTTGGAATTGCTTTTAAAACTGCTAATCTAGAGGTTGAGAGAGGATAGAGGAATTCCTAGTGTAGAGGTGAT
Mper      AGTTAAAAGTGAAATCCCAAGGCTCAACTTTGGAATTGCTTTTAAAACTGCTAACCTAGAGATTGAAAGAGGATAGAGGAATTCCTAGTGTAGAGGTGAA
Bpah      AGTTAAAAGTGAAATCCCAAAGCTTAACTTTGGAATTGCTTTTAAAACTGTTAATCTAGAGGTTGAAAGAGGATAGAGGAATTCCTAGTGTAGAGGTGAT
Kfla      AGTTAAAAGTGAAATCCCAAAGCTCAACTTTGGAATTGCTTTTAAAACTGCTAACCTAGAGATTGAAAGAGGATAGAGGAATTCCTAGTGTAGAGGTGAA
Fcan      AGTTAAAAGTGAAATCCCAAGGCTCAACCTTGGAATTGCTTTTAAAACTGCTAGTCTAGAGATTGAAAGAGGATAGAGGAATTCCTAGTGTAGAGGTGAA
Dmel      AGTTAAAAGTGAAATCCCAAGGCTCAACCTTGGAATTGCTTTTAAAACTGCTAATCTAGAGATTGAAAGAGGATAGAGGAATTCCTAGTGTAGAGGTGAA
DsimwRi   AGTTAAAAGTGAAATCCCAAGGCTCAACCTTGGAATTGCTTTTAAAACTGCTAATCTAGAGATTGAAAGAGGATAGAGGAATTCCTAGTGTAGAGGTGAA
Tcor      AGTTAAAAGAGAAATCCCGAGGCTTAACCTTGGAATTGCTTTTAAAACTATTAGCCTAGAGATTGAAAGAGGATAGAGGAATTCCTGATGTAGAGGTAAA
Cpip      AGTTAAAAGTGAAATCCCGAGGCTTAACCTTGGAATTGCTTTTAAAACTATTAATCTAGAGATTGAAAGAGGATAGAGGAATTCCTGATGTAGAGGTAAA
Dcir      AGTTAAAAGTGAAATCCCAAGGCTCAACCTTGGAATTGCTTTTAAAACTGCTAATCTAGAGATTGAAAGAGGATAGAGGAATTCCTAGTGTAGAGGTGAA

          510       520       530       540       550       560       570       580       590
Bmal      ATTCGTAAATATTAGGAGGAACACCAGTGGCGAAGGCGTCTATCTGGTTCAAATCTGACGCTGAGGCGCGAAAGCGTGGGGAGCAAACAGGATTAGATAC
Ovol      ATTCGTAAATATTAGGAGGAACACCAGTGGCGAAGGCGTCTATCTGGTTCAAATCTGACGCTGAGGTGCGAAAGCGTGGGGAGCAAACAGGATTAGATAC
Ogut      ATTCGTAAATATTAGGAGGAACACCAGTGGCGAAGGCGTCTATCTGGTTCAAATCTGACGCTGAGGTGCGAAAGCGTGGGGAGCAAACAGGATTAGATAC
Dimm      ATTCGTAAATATTAGGAGGAACACCAGTGGCGAAGGCGTCTATCTGGTTCAAATCTGACGCTGAGGTGCGAAAGCGTGGGGAGCAAACAGGATTAGATAC
Lsig      ATTCGTAAATATTAGGAGGAACACCAGTGGCGAAGGCGTCTATCTGGTTCAAATCTGACGCTGAGGCGCGAAGGCGTGGGGAGCAAACAGGATTAGATAC
Mper      ATTCGTAAATATTAGGAGGAACACCAGTGGCGAAGGCGTCTATCTGGTTCAAATCTGACGCTGAGGCGCGAAGGCGTGGGGAGCAAACAGGATTAGATAC
Bpah      ATTCGTAAATATTAGGAGGAACACCAGTGGCGAAGGCGTCTATCTGGTTCAAATCTGACNCTGANGNNCNAAAGCNTGGGGAGCAAACAGGATTAGATNC
Kfla      ATTCGTAAATATTAGGAGGAACACCAGTGGCGAAGGCGTCTATCTGGTTCAAATCTGACGCTGAGGCGCGAAGGCGTGGGGAGCAAACAGGATTAGATAC
Fcan      ATTCGTAAATATTAGGAGGAACACCAGTGGCGAAGGCGTCTATCTGGTTCAAATCTGACGCTGAGGCGCGAAGGCGTGGGGAGCAAACAGGATTAGATAC
Dmel      ATTCGTAAATATTAGGAGGAACACCAGTGGCGAAGGCGTCTATCTGGTTCAAATCTGACGCTGAGGCGCGAAGGCGTGGGGAGCAAACAGGATTAGATAC
DsimwRi   ATTCGTAAATATTAGGAGGAACACCAGTGGCGAAGGCGTCTATCTGGTTCAAATCTGACGCTGAGGCGCGAAGGCGTGGGGAGCAAACAGGATTAGATAC
Tcor      ATTCGTAAATATTAGGAGGAACACCAGTGGCGAAGGCGTCTATCTGGTTCAAATCTGACGCTGAAGCGCGAAGGCNTGGGGAGCAAACAGGATTAGATAC
Cpip      ATTCGTAAATATTAGGAGGAACACCAGTGGCGAAGG-GTCTATCTGGTTCAAATCTGACGCTGAAGC-CGAAGGCGTGGGGAGCAAACAGGATTAGATAC
Dcir      ATTCGTAAATATTA-GAGGAACACCAGTGGCGAA-GCGTCTATCTGGTTCAAATCTGACGCTGAGGCGCGAAGGCGTGGGGAGCAAACAGGATTAGATAC

          610       620       630       640
Bmal      CCTGGTAGTCCACGCTGTAAACGATGAATGTTAAATATGGGAA
Ovol      CCTGGTAGTCCACGCTGTAAACGATGAATGTTAAATATGGGGA
Ogut      CCTGGTAGTCCACGCTGTAAACGATGAATGTTAAATATGGGGA
Dimm      CCTGGTAGTCCACGCTGTAAACGATGAATGTTAAATATGGGAA
Lsig      CCTGGTAGTCCACGCTGTAAACGATGAATGTTAAATATGGGAA
Mper      CCTGGTAGTCCACGCTGTAAACGATGAATGTTAAATATGGGGA
Bpah      CCTGGTANTCCACGCTGTAAACGATGAATGTTAAATATGGGAA
Kfla      CCTGGTAGTCCACGCTGTAAACGATGAATGTTAAATATGGGAA
Fcan      CCTGGTAGTCCACGCTGTAAACGATGAATGTTAAATATGGGAA
Dmel      CCTGGTAGTCCACGCTGTAAACGATGAATGTTAAATATGGGAA
DsimwRi   CCTGGTAGTCCACGCTGTAAACGATGAATGTTAAATATGGGAA
Tcor      CCTGGTAGTCCACGCTGTAAACGATGAATGTTAAATATGGGAA
Cpip      CCTGGTAGTCCACGCTGTAAACGATGAATGTTAAATATGGGGA
Dcir      CCTGGTAGTCCACGCTGTAAACGATGAATGTT-AATATGGGGA
```
