# Supplementary material for: Absence of Wolbachia endobacteria in the non-filariid nematodes Angiostrongylus cantonensis and A. costaricensis
Source: Parasit Vectors. 2008 Sep 18;1:31. doi: 10.1186/1756-3305-1-31 (PMC2565651; doi:10.1186/1756-3305-1-31)
Supplement: Additional file 1 — Wolbachia 16S multiple sequence alignment – A. cantonensis. A multiple sequence alignment of the 16S sequence attributed to Wolbachia from A. cantonensis and corresponding 16S fragments from Wolbachia from diverse arthropod and nematode hosts. [file 1756-3305-1-31-S1.pdf]

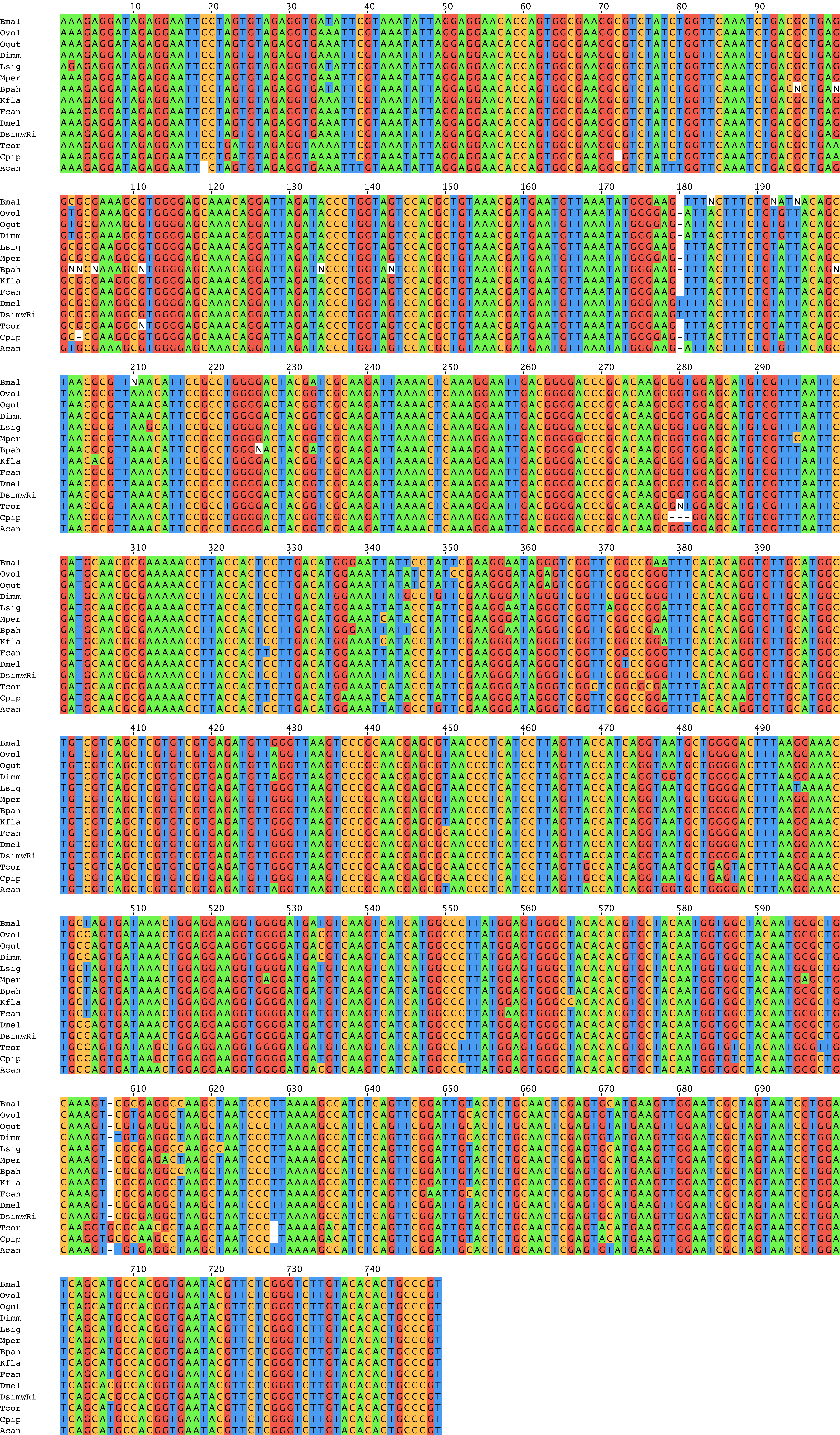
Bmal
Ovol
Ogut
Dimm
Lsig
Mper
Bpah
Kfla
Fcan
Dmel
DsimwRi
Tcor
Cpip
Acan
10
20
30
40
50
60
70
80
90
110
120
130
140
150
160
170
180
190
210
220
230
240
250
260
270
280
290
310
320
330
340
350
360
370
380
390
410
420
430
440
450
460
470
480
490
510
520
530
540
550
560
570
580
590
610
620
630
640
650
660
670
680
690
710
720
730
740
