# Supplementary material for: Absence of Wolbachia endobacteria in the non-filariid nematodes Angiostrongylus cantonensis and A. costaricensis
Source: Parasit Vectors. 2008 Sep 18;1:31. doi: 10.1186/1756-3305-1-31 (PMC2565651; doi:10.1186/1756-3305-1-31)
Supplement: Additional file 3 — Wolbachia ftsZ multiple sequence alignment. A multiple sequence alignment of the ftsZ sequence attributed to Wolbachia from A. cantonensis and corresponding ftsZ fragments from Wolbachia from diverse arthropod and nematode hosts. [file 1756-3305-1-31-S3.pdf]

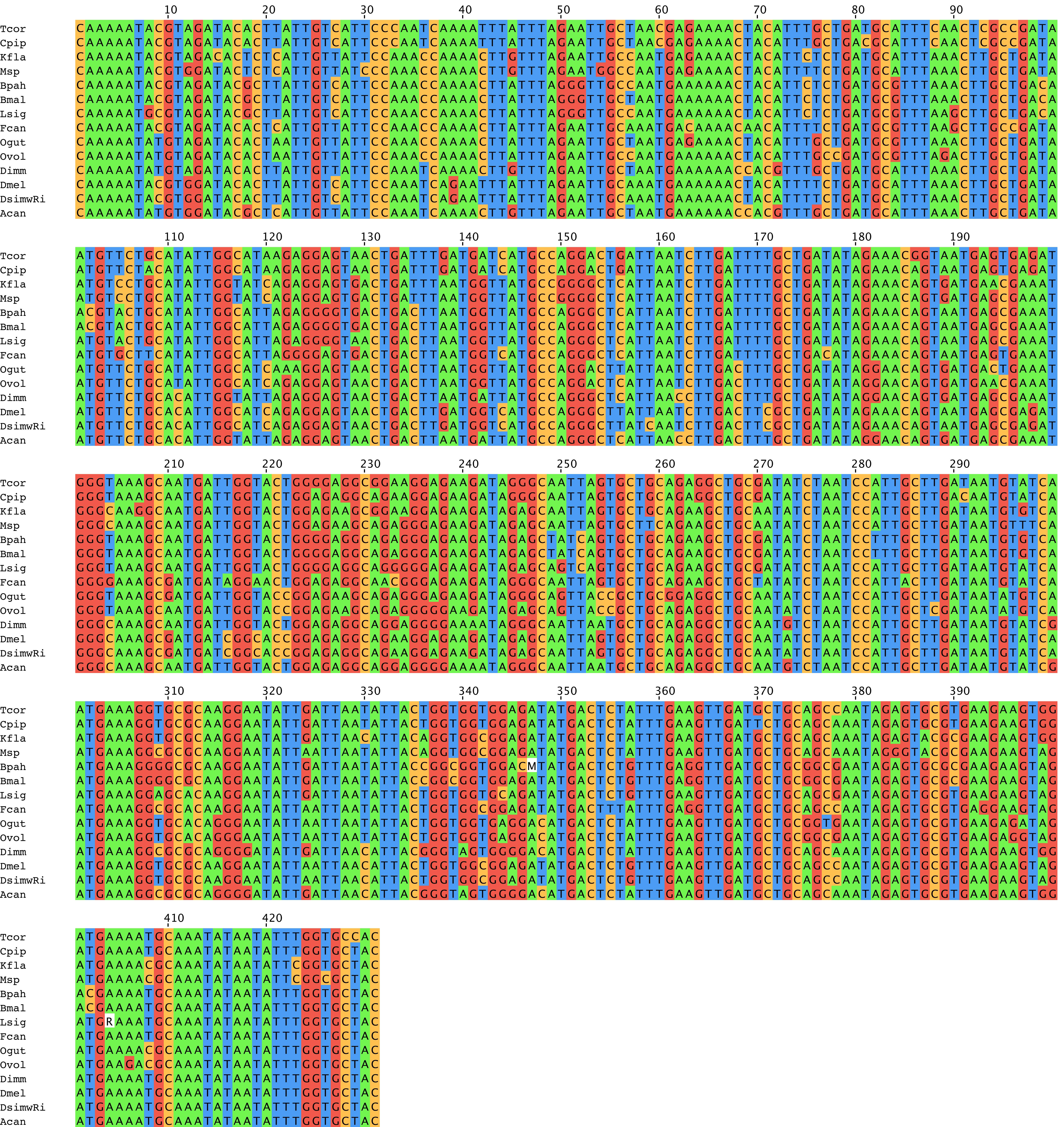
10 20 30 40 50 60 70 80 90
Tcor CAAAAATACGTAGATACACTTATTGTCATTCCCAATCAAAATTTATTTAGAATTGCTAACGAGAAAACTACATTTGCTGATGCATTTCAACTCGCCGATA
Cpip CAAAAATACGTAGATACACTTATTGTCATTCCCAATCAAAATTTATTTAGAATTGCTAACGAGAAAACTACATTTGCTGACGCATTTCAACTCGCCGATA
Kfla CAAAAATACGTAGACACTCTCATTGTTATTCCAAACCAAAACTTGTTTAGAATTGCCAATGAGAAAACTACATTCTCTGATGCATTTAAACTCGCTGATA
Msp CAAAAATACGTGGATACTCTCATTGTTATCCCAAACCAAAACTTGTTTAGAATGGCCAATGAGAAAACTACATTTTCTGATGCATTTAAACTTGCTGATA
Bpah CAAAAATACGTAGATACGCTTATTGTCATTCCAAACCAAAACTTATTTAGGGTTGCCAATGAAAAAACTACATTCTCTGATGCGTTTAAACTTGCTGACA
Bmal CAAAAATACGTAGATACGCTTATTGTCATTCCAAACCAAAACTTATTTAGGGTTGCTAATGAAAAAACTACATTCTCTGATGCGTTTAAACTTGCTGACA
Lsig CAAAAATGCGTAGATACGCTTATTGTCATTCCAAACCAAAACTTATTTAGGGTTGCCAATGAAAAAACTACATTCTCTGATGCGTTTAAGCTTGCTGACA
Fcan CAAAAATACGTAGATACACTCATTGTTATTCCAAACCAAAACTTATTTAGAATTGCAAATGACAAAACAACATTTTCTGATGCGTTTAAGCTTGCCGATA
Ogut CAAAAATATGTAGATACACTAATTGTTATTCCAAACCAAAACTTATTTAGAATTGCTAATGAGAAAACTACATTTGCTGATGCGTTTAAACTTGCTGATA
Ovol CAAAAATATGTAGATACACTAATTGTTATTCCAAACCAAAACTTATTTAGAATTGCCAATGAAAAAACTACATTTGCCGATGCGTTTAGACTTGCTGATA
Dimm CAAAAATATGTAGATACACTTATTGTTATTCCAAATCAAAACTTGTTTAGAATTGCTAATGAAAAAACCACGTTTGCTGATGCATTTAAACTTGCTGATA
Dmel CAAAAATACGTGGATACACTTATTGTCATTCCAAATCAGAATTTATTTAGAATTGCAAATGAAAAAACTACATTTTCTGATGCATTTAAACTTGCTGATA
DsimwRi CAAAAATACGTGGATACACTTATTGTCATTCCAAATCAGAATTTATTTAGAATTGCAAATGAAAAAACTACATTTTCTGATGCATTTAAACTTGCTGATA
Acan CAAAAATATGTGGATACGCTCATTGTTATTCCAAATCAAAACTTGTTTAGAATTGCTAATGAAAAAACCACGTTTGCTGATGCATTTAAACTTGCTGATA
110 120 130 140 150 160 170 180 190
Tcor ATGTTCTGCATATTGGCATAAGAGGAGTAACTGATTTGATGATCATGCCAGGACTGATTAATCTTGATTTTGCTGATATAGAAACGGTAATGAGTGAGAT
Cpip ATGTTCTACATATTGGCATAAGAGGAGTAACTGATTTGATGATCATGCCAGGACTGATTAATCTTGATTTTGCTGATATAGAAACAGTAATGAGTGAGAT
Kfla ATGTCCTGCATATTGGTATCAGAGGAGTGACTGATTTAATGGTTATGCCGGGGCTCATTAATCTTGATTTTGCTGATATAGAAACAGTGATGAACGAAAT
Msp ATGTCCTGCATATTGGTATCAGAGGGGTGACTGATTTAATGGTTATGCCGGGGCTCATTAATCTTGATTTTGCTGATATAGAAACAGTGATGAGCGAAAT
Bpah ACGTACTGCATATTGGCATTAGAGGGGTGACTGACTTAATGGTTATGCCAGGGCTCATTAATCTTGATTTTGCTGATATAGAAACAGTAATGAGCGAAAT
Bmal ACGTACTGCATATTGGCATTAGAGGGGTGACTGACTTAATGGTTATGCCAGGGCTCATTAATCTTGATTTTGCTGATATAGAAACAGTAATGAGCGAAAT
Lsig ATGTACTGCATATTGGCATTAGAGGGGTAACTGACTTAATGGTTATGCCAGGGCTCATTAATCTTGATTTTGCTGATATAGAAACAGTAATGAGCGAAAT
Fcan ATGTGCTTCATATTGGCATTAGGGGAGTGACTGACTTAATGGTCATGCCAGGGCTCATTAATCTTGATTTTGCTGACATAGAAACAGTAATGAGTGAAAT
Ogut ATGTTCTGCATATTGGCATCAAAGGAGTAACTGACTTAATGGTTATGCCAGGACTTATTAATCTTGACTTTGCTGATATAGGAACAGTGATGACTGAAAT
Ovol ATGTTCTGCATATTGGCATCAGAGGAGTAACTGACTTAATGGTTATGCCAGGACTCATTAATCTTGACTTTGCTGATATAGGAACAGTGATGAACGAAAT
Dimm ATGTTCTGCACATTGGTATTAGAGGAGTAACTGACTTAATGATTATGCCAGGGCTCATTAACCTTGACTTTGCTGATATAGGAACAGTGATGAGCGAAAT
Dmel ATGTTCTGCACATTGGCATCAGAGGAGTAACTGACTTGATGGTCATGCCAGGGCTTATTAATCTTGACTTCGCTGATATAGAAACAGTAATGAGCGAGAT
DsimwRi ATGTTCTGCACATTGGCATCAGAGGAGTAACTGACTTGATGGTCATGCCAGGGCTTATCAATCTTGACTTCGCTGATATAGAAACAGTAATGAGCGAGAT
Acan ATGTTCTGCACATTGGTATTAGAGGAGTAACTGACTTAATGATTATGCCAGGGCTCATTAACCTTGACTTTGCTGATATAGGAACAGTGATGAGCGAAAT
210 220 230 240 250 260 270 280 290
Tcor GGGTAAAGCAATGATTGGTACTGGGGAGGCGGAAGGAGAAGATAGGGCAATTAGTGCTGCAGAGGCTGCGATATCTAATCCATTGCTTGATAATGTATCA
Cpip GGGTAAAGCAATGATTGGTACTGGAGAGGCAGAAGGAGAAGATAGGGCAATTAGTGCTGCAGAGGCTGCGATATCTAATCCATTGCTTGACAATGTATCA
Kfla GGGCAAGGCAATGATTGGTACTGGAGAAGCGGAAGGAGAAGATAGAGCAATTAGTGCTGCAGAAGCTGCAATATCTAATCCATTGCTTGATAATGTGTCA
Msp GGGCAAAGCAATGATTGGTACTGGAGAAGCAGAGGGAGAAGATAGAGCAATTAGTGCTTCAGAAGCTGCAATATCTAATCCATTGCTTGATAATGTTTCA
Bpah GGGTAAAGCAATGATTGGTACTGGGGAGGCAGAGGGAGAAGATAGAGCTATCAGTGCTGCAGAAGCTGCGATATCTAATCCTTTGCTTGATAATGTGTCA
Bmal GGGTAAAGCAATGATTGGTACTGGGGAGGCAGAGGGAGAAGATAGAGCTATCAGTGCTGCAGAAGCTGCGATATCTAATCCTTTGCTTGATAATGTGTCA
Lsig GGGTAAAGCAATGATTGGTACTGGGGAGGCAGGGGGAGAAGATAGAGCAGTCAGTGCTGCAGAAGCTGCGATATCTAATCCATTGCTTGATAATGTATCA
Fcan GGGGAAAGCGATGATAGGAACTGGAGAGGCAACGGGAGAAGATAGGGCAATTAGTGCTGCAGAAGCTGCTATATCTAATCCATTACTTGATAATGTATCA
Ogut GGGTAAAGCGATGATTGGTACCGGAGAAGCAGAGGGAGAAGATAGGGCAGTTACCGCTGCGGAGGCTGCAATATCTAATCCATTGCTTGATAATATGTCA
Ovol GGGTAAAGCAATGATTGGTACCGGAGAAGCAGAGGGGGAAGATAGAGCAGTTACCGCTGCAGAGGCTGCAATATCTAATCCATTGCTCGATAATATGTCA
Dimm GGGCAAAGCAATGATTGGTACTGGAGAGGCAGGAGGGGAAAATAGGGCAATTAATGCTGCAGAGGCTGCAATGTCTAATCCATTGCTTGATAATGTATCG
Dmel GGGCAAAGCGATGATCGGCACCGGAGAGGCAGAAGGAGAAGATAGAGCAATTAGTGCTGCAGAGGCTGCAATATCTAATCCATTGCTTGATAATGTATCA
DsimwRi GGGCAAAGCGATGATCGGCACCGGAGAGGCAGAAGGAGAAGATAGAGCAATTAGTGCTGCAGAGGCTGCAATATCTAATCCATTGCTTGATAATGTATCA
Acan GGGCAAAGCAATGATTGGTACTGGAGAGGCAGGAGGGGAAAATAGGGCAATTAATGCTGCAGAGGCTGCAATGTCTAATCCATTGCTTGATAATGTATCG
310 320 330 340 350 360 370 380 390
Tcor ATGAAAGGTGCGCAAGGAATATTGATTAATATTACTGGTGGTGGAGATATGACTCTATTTGAAGTTGATGCTGCAGCCAATAGAGTGCGTGAAGAAGTGG
Cpip ATGAAAGGTGCGCAAGGAATATTGATTAATATTACTGGTGGTGGAGATATGACTCTATTTGAAGTTGATTCTGCAGCAAATAGAGTGCGTGAAGAAGTGG
Kfla ATGAAAGGTGCGCAAGGAATATTGATTAACATTACAGGTGGCGGAGATATGACTCTATTTGAAGTTGATGCTGCAGCAAATAGAGTACGCGAAGAAGTGG
Msp ATGAAAGGCGCGCAAGGAATATTAATTAATATTACAGGTGGCGGAGATATGACTCTATTTGAAGTTGATGCTGCAGCAAATAGGGTACGCGAAGAAGTAG
Bpah ATGAAAGGGGCGCAAGGAATATTGATTAATATTACCGGCGGTGGACMTATGACTCTGTTTGAGGTTGATGCTGCGGCGAATAGAGTGCGCGAAGAAGTAG
Bmal ATGAAAGGGGCGCAAGGAATATTGATTAATATTACCGGCGGTGGAGATATGACTCTGTTTGAGGTTGATGCTGCGGCGAATAGAGTGCGCGAAGAAGTAG
Lsig ATGAAAGGAGCGCAAGGAATATTGATTAATATTACTGGTGGTGCAGATATGACTCTGTTTGAAGTTGATGCTGCAGCGAATAGAGTGCGTGAAGAAGTAG
Fcan ATGAAAGGCGCACAAGGAATATTAATTAATATTACTGGTGGCGGAGATATGACTTTATTTGAGGTTGATGCTGCAGCCAATAGAGTGCGTGAGGAAGTAG
Ogut ATGAAAGGTGCACAGGGAATATTAATTAATATTACTGGTGGTGAGGACATGACTCTATTTGAAGTTGATGCTGCGGTGAATAGAGTGCGTGAAGAGATAG
Ovol ATGAAAGGTGCACAGGGAATATTAATTAATATTACTGGTGGTGAGGACATGACTCTATTTGAAGTTGATGCTGCGGCGAATAGAGTGCGTGAAGAGGTAG
Dimm ATGAAAGGCGCGCAGGGGATATTGATTAACATTACGGGTAGTGGGGACATGACTCTATTTGAAGTTGATGCTGCAGCAAATAGAGTGCGTGAAGAAGTGG
Dmel ATGAAAGGTGCGCAAGGAATATTAATTAACATTACTGGTGGCGGAGATATGACTCTGTTTGAAGTTGATGCTGCAGCCAATAGAGTGCGTGAAGAAGTAG
DsimwRi ATGAAAGGTGCGCAAGGAATATTAATTAACATTACTGGTGGCGGAGATATGACTCTGTTTGAAGTTGATGCTGCAGCCAATAGAGTGCGTGAAGAAGTAG
Acan ATGAAAGGCGCGCAGGGGATATTGATTAACATTACGGGTAGTGGGGACATGACTCTATTTGAAGTTGATGCTGCAGCAAATAGAGTGCGTGAAGAAGTGG
410 420
Tcor ATGAAAATGCAAATATAATATTTGGTGCCAC
Cpip ATGAAAATGCAAATATAATATTTGGTGCTAC
Kfla ATGAAAACGCAAATATAATATTCGGTGCTAC
Msp ATGAAAACGCAAATATAATATTCGGCGCTAC
Bpah ACGAAAATGCAAATATAATATTTGGTGCTAC
Bmal ACGAAAATGCAAATATAATATTTGGTGCTAC
Lsig ATGRAAATGCAAATATAATATTTGGTGCTAC
Fcan ATGAAAATGCAAATATAATATTTGGTGCTAC
Ogut ATGAAAACGCAAATATAATATTTGGTGCTAC
Ovol ATGAAGACGCAAATATAATATTTGGTGCTAC
Dimm ATGAAAATGCAAATATAATATTTGGTGCTAC
Dmel ATGAAAATGCAAATATAATATTTGGTGCTAC
DsimwRi ATGAAAATGCAAATATAATATTTGGTGCTAC
Acan ATGAAAATGCAAATATAATATTTGGTGCTAC
